# Clinical Analysis of Intestinal Tuberculosis: A Retrospective Study

**DOI:** 10.3390/jcm12020445

**Published:** 2023-01-05

**Authors:** Jiaqi Zeng, Guanzhou Zhou, Fei Pan

**Affiliations:** 1 Department of Gastroenterology and Hepatology, The First Medical Center, Chinese PLA General Hospital, Beijing 100853, China; 2 Chinese PLA Medical School, Beijing 100853, China; 3 Medical School, Nankai University, Tianjin 300071, China

**Keywords:** intestinal tuberculosis, clinical manifestations, laboratory findings, radiological results, endoscopic image, histological and microbiological findings

## Abstract

Purpose: This study aimed to summarize and analyze the clinical data of intestinal tuberculosis (ITB) in order to provide guidance for accurate diagnosis and treatment of ITB. Methods: This study consecutively included patients with ITB who were admitted to our hospital from 2008 to 2021 and retrospectively analyzed their clinical features. Results: Forty-six patients were included. The most common clinical symptom was weight loss (67.4%). Seventy percent of 20 patients were positive for tuberculin skin test; 57.1% of 14 patients were positive for mycobacterium tuberculosis specific cellular immune response test, while 84.6% of 26 patients were positive for tuberculosis infection T cell spot test. By chest computed tomography (CT) examination, 25% and 5.6% of 36 patients were diagnosed with active pulmonary tuberculosis and with inactive pulmonary tuberculosis, respectively. By abdominal CT examination, the most common sign was abdominal lymph node enlargement (43.2%). Forty-two patients underwent colonoscopy, and the most common endoscopic manifestation was ileocecal ulcer (59.5%), followed by colonic ulcer (35.7%) and ileocecal valve deformity (26.2%). ITB most frequently involved the terminal ileum/ileocecal region (76.1%). Granulomatous inflammation with multinucleated giant cells and caseous necrosis was found via endoscopic biopsies, the ultrasound-guided percutaneous biopsy of enlarged mesentery lymph nodes, and surgical interventions. The acid-fast bacilli were discovered in 53.1% of 32 samples. Twenty-one cases highly suspected of ITB were confirmed after responding to empiric anti-tuberculosis therapy. Conclusions: It was necessary to comprehensively analyze clinical features to make an accurate diagnosis of ITB and aid in distinguishing ITB from diseases such as Crohn’s disease and malignant tumors.

## 1. Introduction

Nowadays, tuberculosis, a common infectious disease, remains a public health problem of global concern [1]. Extrapulmonary tuberculosis (EPTB) accounts for a significant proportion of the population, among which TB can involve any part of the gastrointestinal tract. Gastroduodenal TB can mimic esophageal cancer, esophageal ulcers, submucosal tumors, gastric erosions, ulcerated gastric masses, and gastric outlet obstruction [2,3,4].

Intestinal tuberculosis (ITB) can be a primary infection or a secondary infection, usually secondary to pulmonary tuberculosis [5]. A large-scale multi-center study in China showed that among patients with pulmonary tuberculosis complicated with EPTB, 72.04% were complicated with ITB. Accordingly, pulmonary tuberculosis should be considered when a diagnosis of ITB is made [6].

ITB is thought to be more common in young and middle-aged patients. Studies from Nepal [7] and France [8] suggested a higher proportion of women with ITB, whereas other studies suggested an equal or a higher proportion of men with ITB [9,10]. Previous studies suggested that risk factors of ITB included low social status; immunodeficiency, such as HIV infection; organ transplantation; and the use of immunosuppressants [2].

A diagnosis of ITB is rather challenging due to the protean clinical manifestations. The confirmed diagnosis depends on histological and microbiological examinations, the sensitivity of which is relatively low. Missed diagnosis, misdiagnosis, and delayed diagnosis and treatment can lead to severe complications and even mortality. Studies have shown that the mortality rate of ileocecal tuberculosis can be as high as 19–38% [2]. Additionally, ITB shared a variety of clinical manifestations with diseases, especially inflammatory bowel diseases such as Crohn’s disease (CD), that require careful differential diagnosis [11].

Therefore, it is important to improve the ability to make an early diagnosis. This study aimed to summarize and analyze the clinical data of ITB in order to provide guidance for the early diagnosis and treatment of ITB.

## 2. Materials and Methods

### 2.1. Study Design

This study was a retrospective study and was approved by the ethics committee of Chinese PLA General Hospital. Patients with ITB admitted to the First Medical Center of Chinese PLA General Hospital from 2008 to 2021 were consecutively included. Inclusion criteria included at least one of the following: (1) typical histological findings: caseous or necrotizing granuloma; (2) tissue culture positive for mycobacterium tuberculosis; (3) tissue positive for acid fast bacteria (AFB); (4) mycobacterium tuberculosis detected by PCR; and (5) the resolution of symptoms after 6 weeks of standard anti-tuberculosis therapy (ATT). Exclusion criteria were as follows: (1) patients with incomplete clinical information; (2) patients lost to follow-up; and (3) a diagnosis of ITB could not be confirmed.

### 2.2. Data Extraction

Data extraction and input were carried out by two researchers, and the inconsistent results were re-input. The following information was collected: sociodemographic patterns, risk factors, course of disease, clinical manifestations, laboratory indicators, radiological findings (including chest computed tomography (CT), abdominal ultrasound, and abdominal CT), endoscopic findings, histological findings, microbiological findings, and treatment and its results.

### 2.3. Statistical Analysis

Statistical descriptive analysis was mainly used. Quantitative data are presented as mean ± standard deviation or median, and qualitative data are presented as numbers and percentages. The SPSS 26.0 software package was used for data analysis.

## 3. Results

### 3.1. Patient Recruitment and Characteristics

Of the 109 patients initially diagnosed with ITB, 12 were lost to follow-up and 51 were excluded due to insufficient evidence for final diagnosis or a diagnosis of other diseases (e.g., CD). Eventually, a total of 46 patients with ITB were included in the study. The demographic characteristics are shown in Table 1. Among the patients, risk factors of ITB included compromised immune systems, contact with tuberculosis patients, a previous history of tuberculosis, and unclean foods, as shown in Table 2.

### 3.2. Clinical Manifestations

The median time from symptom onset to admission to our hospital was 5 months. Clinical manifestations were collated in Table 3. The most common clinical symptoms were weight loss (67.4%), followed by abdominal pain (65.2%), fever (39.1%), abdominal bloating (30.4%), and diarrhea (21.7%). Abdominal tenderness was the most common physical sign (34.8%), followed by abdominal mass (15.2%) and abdominal distension (4.3%).

### 3.3. Laboratory Tests

The laboratory findings were collated in Table 4. Of the patients, 68.4% (26/38) had elevated C-reactive protein (CRP), 76.5% (26/34) had elevated erythrocyte sedimentation rate (ESR), 71.0% (22/31) had elevated D-dimer, and 91.9% (12/15 in females, 22/22 in males) had decreased hemoglobin (Hb). The positive rate of fecal occult blood test was 32.5% (13/40).

Among 20 patients, 14 (70%) were positive for tuberculin skin test (TST), of which 9 (64.3%) were strongly positive. Among 14 patients, 8 (57.1%) were positive for the mycobacterium-tuberculosis-specific cellular immune response (A.TB, ELISA) test. Among 26 patients, 22 (84.6%) were positive for the tuberculosis infection T cell spot test (T-SPOT).

### 3.4. Radiological Examination

Chest CT examination was performed in 36 patients, with 9 (25%) having active pulmonary tuberculosis and 2 (5.6%) having inactive pulmonary tuberculosis. The radiological features of active pulmonary tuberculosis mainly manifested as follows: multiple nodules, patchy shadow and consolidation in the lung, blurred lesion boundaries, calcification, cavity formation, and multiple small subpleural nodules, whereas the radiological features of inactive pulmonary tuberculosis mainly manifested as calcification and a fibrous streak shadow in the lung.

Abdominal ultrasonography was performed in 19 patients, with 1 patient (5.3%) having ascites, 1 patient (5.3%) having increased intestinal wall thickness of the terminal ileum/ileocecal region, 2 patients (10.5%) having hypoechoic intestinal nodules, and 1 patient (5.3%) having irregular intestinal wall thickness in the ascending colon. A total of 37 patients underwent abdominal CT examination, and the results are shown in Table 5. The thickening of the terminal ileum/ileocecal region can be characterized by irregular thickening, segmental thickening, soft tissue mass shadow, and annular enhancement. The thickening of the colon wall can be manifested as mass, uneven thickening, leaping and annular thickening, irregular mass and low-density shadow, and uneven and even enhancement with an enhanced CT examination. The thickening of the intestinal wall may appear as segmental thickening.

### 3.5. Endoscopy, Histological Examination, and Microbiological Examination

**Figure 1:**
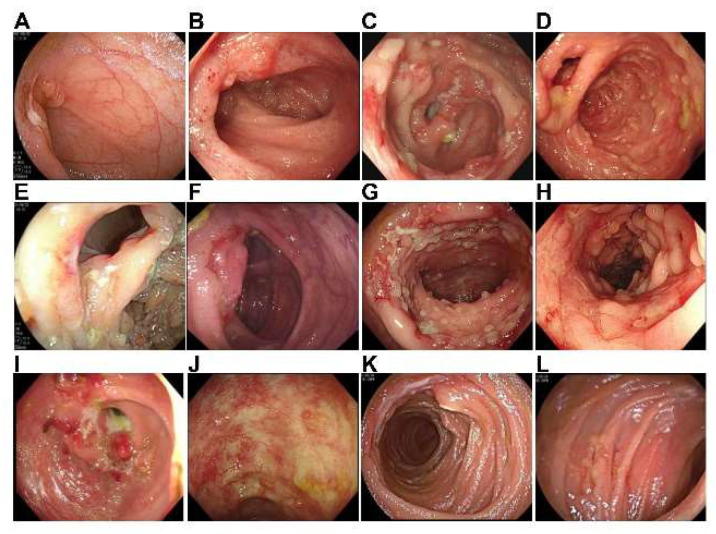
Endoscopic images of patients with ITB. (**A**) Ulcer in the terminal ileal; (**B**) inflammation and erosion in the terminal ileal; (**C**) ulcer in the ileocecal region; (**D**) inflammation and erosion in the ileocecal region; (**E**) ileocecal valve deformation; (**F**) inflammation and erosion in the ileocecal valve; (**G**) pseudopolyps and colonic ulcer; (**H**) colonic inflammation; (**I**) colonic stricture and colonic mass; (**J**) colonic scar; (**K**) jejunal ulcer; and (**L**) small intestinal inflammation.

Forty-two patients underwent colonoscopy, and the results are presented in Table 6. The representative endoscopic images are shown in Figure 1. The ulcers in the terminal ileum/ileocecal region included large ulcers, irregular ulcers, quasi-circular ulcers of different sizes and depths, scattered punctiform, flake and strip ulcers, scattered deep ulcers, peripherical deep ulcers, annular ulcers, segmental ulcers, and ulcers with a cobblestone appearance. Ileocecal valve deformation manifested as a patulous ileocecal valve and a constricted ileocecal valve. Colonic ulcers manifested as quasi-circular ulcers of different sizes and depths, scattered punctiform ulcers, scattered deep ulcers, and annular ulcers.

The most common site of involvement was ileocecal area (76.1%), followed by the ascending colon (43.5%), descending colon (8.7%), transverse colon (13.0%), jejunum (8.6%), and sigmoid colon (2.2%).

Thirty-six patients underwent endoscopic biopsies, and the histological manifestations included chronic granulomatous inflammation (44.4%) and hyperplasia of epithelioid cells (5.6%). Nine patients underwent surgical prevention due to intestinal obstruction, unclear diagnosis, etc. Postoperative histology included granulomatous inflammation with multinucleated giant cells (66.7%) and caseous necrosis (33.3%). One patient underwent an ultrasound-guided percutaneous biopsy of enlarged mesenteric lymph nodes, which manifested as epithelioid cell granuloma and multinucleated giant cells.

AFB were detected in 17 of the 32 patients via acid-fast staining (53.1%).

### 3.6. Empiric Antituberculosis Therapy

Twenty-one cases of highly suspected ITB, which could not be confirmed by histology and microbiology, underwent empiric ATT, and after treatment, their symptoms disappeared and the intestinal mucosa healed. Of them, 1 patient relapsed after 1 year of treatment and recovered after 2 years of further ATT, while 1 patient had persistent symptoms after 6-month ATT and recovered after 1-year treatment with added amikacin.

## 4. Discussion

This study showed that ITB mainly affected young and middle-aged adults, with a higher proportion of males than females, which was consistent with the results of a large-scale multi-center study in China [6]. Risk factors of ITB in this study included compromised immune systems; diabetes mellitus (DM); chronic renal failure; kidney transplantation; rheumatic diseases; contact with patients with TB; a history of TB; cancer; hypothyroidism; and eating unclean foods. Patients with chronic renal failure, kidney transplantation, and hypothyroidism are more susceptible to TB because of impaired immune function [12]. DM patients are more susceptible to mycobacterium tuberculosis due to compromised immunity, metabolic disorders, and a hyperglycemic microenvironment conducive to bacterial growth and reproduction. The ability of macrophages to clear mycobacterium tuberculosis was decreased in patients with rheumatic diseases due to taking corticosteroids and immunosuppressants [13]. Clinical studies have demonstrated that the risk of TB reactivation is significantly increased in patients with latent TB infection who receive anti-TNF -α therapy [14]. A nationwide population-based cohort study showed that compared with the control group, patients with cancer had a higher incidence of TB, which was related to mucosal barrier impairment and compromised immunity due to the tumor itself and the treatment of radiotherapy and chemotherapy [15].

It was previously thought that people with TB often had poor socio-economic conditions, including poor quality of life and poor education [16]. However, this study suggested that there was no remarkable difference in occupational composition. This may be associated with the presence of other risk factors of ITB in high-income people, such as DM, organ transplantation, and use of immunosuppressants.

The most common symptoms in patients with ITB were weight loss and abdominal pain. CD can also present with weight loss and abdominal pain. However, low-grade fever and night sweats are more common in patients with ITB, while haematochezia are more common in patients with CD [17]. 

CRP and ESR, being inflammatory indicators, can increase in a variety of diseases, while decreased Hb and increased D-dimer are also seen in a variety of diseases. As this study is a descriptive study and lacks a control group, the diagnostic specificity cannot be analyzed. Previous studies suggested that D-dimer was commonly elevated in patients with active pulmonary TB and TB with HIV infection [18]. In this study, 25% of patients with active pulmonary TB were not co-infected with HIV, so routine testing of D-dimer in patients with ITB may indicate the pulmonary TB on the one hand and assist in the diagnosis of ITB on the other hand. As it is sometimes difficult to distinguish ITB from gastrointestinal neoplasms, tumor markers were included for analysis, and the results were within the normal range.

The diagnostic sensitivity of the T-SPOT test and TST was relatively high, 84.6% and 70%, respectively. Previous studies have shown that the specificity of the two was approximately 65% [19] and 93% [20], respectively. It should be noted that the results of these two tests are related to the immune status of the patients. The results of TST of patients who have been vaccinated with BCG can be falsely positive, while those of patients who have received immunosuppressants can be falsely negative [21]. For those with an impaired immune response such as malnutrition, the use of immunosuppressants, and advanced age, the results of the T-SPOT test may be falsely negative [22]. There are currently two types of interferon-gamma release assay (IGRA) tests, including A.TB, ELISA, and ELISPOT (T-SPOT.TB). Previous studies have shown no statistical difference in sensitivity and specificity between the two tests. The results of this study showed that the diagnostic sensitivity of A.TB and ELISA was 57.1%. A combination of these two tests can raise the diagnostic efficacy [23]. 

In this study, chest CT imaging showed that 30.6% of patients with ITB were concomitant with pulmonary TB. Therefore, patients with ITB should be routinely screened for pulmonary TB. In addition, imaging findings of TB can be confused with malignant tumors. Nevertheless, the tumor markers of patients with ITB were mostly normal or slightly elevated. Abdominal CT imaging showed a low positive rate of abdominal lymph node calcification, the sign of which was valuable in differentiating CD from ITB. Moreover, the imaging of CD is often segmental, but this can also be present in ITB [24].

ITB most often involves the ileocecal area, probably due to the longer retention time of intestinal contents and the abundance of lymphoid tissue in this region [2]. Mycobacterium tuberculosis penetrates the mucosa and invades submucosal lymphatic tissue, triggering an inflammatory response at this site, followed by subsequent pathology.

Endoscopic manifestations of ITB and CD are difficult to differentiate. The involvement of the left colon, longitudinal ulcers, aphthous ulcers, unlcers with a cobblestone appearance, and segmental lesions are more commonly seen in CD [25,26], whereas involvement of the ascending colon, ileocecal valve deformation, pseudopolyp, and scar formation are more indicative of ITB.

About half of the histological results obtained by colonoscopy suggested ITB. When the diagnosis was unclear and patients presented with intestinal obstruction or malignant signs, a considerable proportion of patients underwent surgical intervention. In order to reduce the surgical intervention, a comprehensive analysis of the examination results should be performed, and more frequent endoscopic biopsy or an increased number of biopsy samples should be considered to assist in diagnosis [27]. For patients with abdominal lymph-node enlargement, ultrasound-guided percutaneous biopsy can be considered after careful evaluation. In this study, to make a confirmed diagnosis, ATT was required for 46% of patients. For patients with a poor treatment response yet highly suspected of ITB, measures such as changing anti-tuberculosis drugs and increasing the dose should be considered.

ITB should be differentiated from malignant tumors and CD. The misdiagnosis of cancer results in unnecessary surgical resection, which imposes physical and mental burdens on patients. Hence, the possibility of ITB should be considered when cancer is suspected yet without histological evidence. The differentiation between ITB and CD is challenging. At present, biological agents are increasingly used to treat CD, which worsens the condition and even causes serious consequences for patients with ITB misdiagnosed as CD [28]. Therefore, empiric ATT should be considered in clinical practice.

Apart from ATT, in clinical practice, surgery is often used as an important adjunct in cases with complications, including bleeding, perforation, stricture, abscess, and fistula, that are refractory to antimicrobial drugs [2,29]. Patients with strictures may receive endoscopic dilation or surgical intervention when they fail to respond to ATT. Compared to surgery, endoscopic intervention is hard to perform in cases with small intestinal strictures, long strictures, and multiple strictures [29].

There are some limitations in this study. Firstly, it is a retrospective single-center study with relatively small sample sizes. Secondly, our hospital is not a specialized tuberculosis hospital, and the patients were transferred to specialized tuberculosis hospitals for further treatment after diagnosis. Thirdly, the evaluation of the therapeutic effect on the patients depended on the patients’ oral statements during follow-up, which may have caused memory bias.

## 5. Conclusions

In summary, it is necessary to comprehensively analyze clinical features to make an accurate diagnosis of ITB. Additionally, it is important to differentiate ITB from diseases such as Crohn’s disease and malignant tumors to avoid the harmful effects of wrong treatment.

## Figures and Tables

**Table 1:** Demographic characteristics of patients with intestinal tuberculosis.

Variable	Value
Age	43.2 ± 15.3
Gender	
Male	67.4%
Female	32.6%
Occupation	
Freelancer	37.0%
Middle-class worker	30.4%
Blue-collar worker	15.2%
Student	8.7%
Farmer	6.5%
Soldier	2.2%

**Table 2:** The risk factors of patients with intestinal tuberculosis. TB: tuberculosis.

Risk Factors	Cases (*n* = 46)	Percent
Immunosuppressive drug use	8	17.4%
Rheumatic disease	8	17.4%
Potential contact with a TB patient	5	10.9%
Previous TB history	4	8.7%
Diabetes mellitus	3	6.5%
Kidney transplantation	2	4.3%
Hypothyroidism	2	4.3%
Chronic renal failure	1	2.2%
Cancer	1	2.2%
Unclean foods	1	2.2%

**Table 3:** Clinical manifestations of patients with intestinal tuberculosis.

Symptoms and Signs	Cases (*n* = 46)	Percent (%)
Weight loss	31	67.4%
Abdominal pain	30	65.2%
Fever	18	39.1%
Abdominal tenderness	16	34.8%
Abdominal bloating	14	30.4%
Diarrhea	10	21.7%
Weakness	8	17.4%
Bloody defecation	8	17.4%
Poor appetite	7	15.2%
Palpable abdominal mass	7	15.2%
Night sweats	5	10.9%
Cough	2	4.3%
Constipation	2	4.3%
Abdominal distension	2	4.3%
Alternating diarrhea and constipation	1	2.2%

**Table 4:** Laboratory findings of patients with intestinal tuberculosis.

Laboratory Indicators	Data	Reference Value
CRP (mg/dL)	2.0 (0.1–19.6)	0–0.8
WBC (/L)	7.5 ± 3.4 × 10^9^	3.5–10 × 10^9^
ESR (mm/h)	38.4 ± 25.9	<20
Hb (g/L)	Male: 118.3 ± 26.5, female: 103.2 ± 18.2	Male: 137–179, female: 116–155
HCT (L/L)	Male: 0.37 (0.18–0.49), female: 0.32 (0.21–0.40)	Male: 0.4–0.52, female: 0.37–0.47
Lymphocyte (%)	23 (1–73)	20–40
Platelet (/L)	282.3 ± 108.5 × 10^9^	100–300 × 10^9^
D-dimer (μg/mL)	0.8 (0.15–5.52)	0–0.5
CEA (μg/L)	1.5 (0.2–7.4)	0–5
CA125 (μ/mL)	18.0 (4.5–237.5)	0.1–35
CA199 (μ/mL)	6.2 (0.6–94.8)	0.1–37
CA153 (μ/mL)	12.9 ± 7.0	0.1–30
CA724 (μ/mL)	1.4 (0.8–10.3)	0.1–10
Serum ferritin (ng/mL)	Male: 292.8 (7.8–2000), female: 148 (47.8–712.1)	Male: 30–400, female: 13–150
Albumin (g/L)	36.6 ± 5.8	35–55

CRP: C-reactive protein; WBC: white blood cell count; ESR: erythrocyte sedimentation rate; Hb: hemoglobin; HCT: red-blood-cell-specific volume; CEA: carcinoembryonic antigen; and CA: carbohydrate antigen.

**Table 5:** Abdominal CT examination findings of patients with intestinal tuberculosis.

Radiological Manifestations	Cases (*n* = 37)	Percent
Enlarged celiac lymph nodules	16	43.2%
Thickening of the terminal ileum/ileocecal region	10	27.0%
Thickening of the colon wall	9	24.3%
Thickening of the small intestinal wall	6	16.2%
Intra-abdominal abscess	1	2.7%
Calcified celiac lymph nodules	1	2.7%

**Table 6:** Endoscopic features of patients with intestinal tuberculosis.

Features	Cases (*n* = 42)	Percent
Ulcer in the terminal ileal /ileocecal region	25	59.5%
Colonic ulcer	15	35.7%
Ileocecal valve deformation	11	26.2%
Pseudopolyps	9	21.4%
Inflammation and erosion in the terminal ileal/ileocecal region	7	16.7%
Stricture	6	14.3%
Colonic inflammation	5	11.9%
Inflammation and erosion in the ileocecal valve	1	2.4%
Scar	1	2.4%
Jejunal ulcer	1	2.4%
Small intestinal inflammation	1	2.4%
Colonic mass	1	2.4%

## Data Availability

Supporting data are available without restrictions from Fei Pan.
